# Linking educational leadership styles to the HR architecture for new teachers in primary education

**DOI:** 10.1186/s40064-016-3378-8

**Published:** 2016-10-07

**Authors:** Eva Vekeman, Geert Devos, Martin Valcke

**Affiliations:** Department of Educational Studies, Faculty of Psychology and Educational Sciences, Ghent University, Henri Dunantlaan 2, 9000 Ghent, Belgium

**Keywords:** Human resource management, New teachers, Instructional leadership, Transformational leadership

## Abstract

This study aims to gain insight in the relationship between principals’ leadership styles and the configuration of different HR practices for new teachers in primary education. Besides the longstanding interest in educational leadership as a key element in teacher and student performance, there is a growing interest in strategic human resource management (SHRM) in the educational sector. However, few educational studies link educational leadership to SHRM. In particular, this study examines the relationship between principals’ instructional and transformational leadership style and principals’ strategic and HR orientation in configuring HR practices for new teachers. Data were gathered using a mixed methods approach, including interviews with 75 principals as well as an online survey of 1058 teachers in Flemish primary education. Qualitative interview data were transformed and analysed together with the quantitative survey data using logistic regression and ANOVA analyses. The results indicate that both instructional and transformational leadership is associated with the strategic orientation of principals. The HR orientation, on the other hand, is not reflected in the principals’ leadership style. Recommendations for further research in this area are discussed.

## Background

For years, researchers have made strides documenting the importance of human resource management (HRM) (e.g. Arthur 1994) and more specifically strategic human resource management (SHRM) for employees’ and organisations' performance (e.g. Wright and Snell 1998). Yet, only recently attention has been given to the importance of (S)HRM in improving and developing schools (Leisink and Boselie 2014; Odden 2011; Runhaar and Runhaar 2012; Smylie and Wenzel 2006). Actually, the awareness has grown that school leaders can have a tremendous effect on student learning through the teachers they hire, how they assign those teachers to classrooms, how they retain teachers and how they create opportunities for teachers to improve (Horng and Loeb 2010). Moreover, research showed that school principals’ organisational management practices—particularly, in the area of SHRM—appear to play a critical role in improving schools (Beteille et al. 2012). At the same time, it has been well recognised that the leadership style of the school principal can strongly influence various elements of the school environment, including teachers’ practice (e.g. Leithwood and Jantzi 2006) and students’ learning (e.g. Robinson et al. 2008). Therefore, it is surprising that scant research exists that integrates (S)HRM and educational leadership.

Also outside the educational field, HRM and leadership were separate research areas for many years (Liu et al. 2003; Vermeeren 2014). Yet, the interest in combining leadership and HRM has grown gradually based on the premise that employees are likely to be influenced by both the HR practices they experience and their supervisor’s leadership style (Purcell and Hutchinson 2007). Moreover, it is appropriate to assume a relationship exists between leadership styles and HRM. Purcell et al. (2009) argue that the way managers undertake their HR duties is linked to leadership behaviour. Furthermore, previous research has emphasised that the behaviour of managers can be explained by their leadership styles (Bass 1990), and managers are likely to implement the HR practices that fit their leadership style (Guest 1987). Yet, only a few studies investigated the direct relationship between supervisors’ leadership styles and HRM (Vermeeren 2014; Vermeeren et al. 2014; Zhu et al. 2005). Zhu et al. (2005) found, for example, that transformational leaders are more likely to adopt human–capital enhancing HRM. Furthermore, studies by Vermeeren and colleagues indicated a transformational leadership style is positively related with the use of commitment-oriented HR practices (Vermeeren 2014) or the amount of HR practices used (Vermeeren et al. 2014). Yet, until today, it is unclear how different leadership styles are related to the configuration of a bundle of HR practices in education. Investigating a bundle of HR practices is important since a growing body of evidence suggests that complementarities or synergies both among an organisation’s different practices as well as between an organisation’s HR practices and its strategy have a reinforcing effect on performance (e.g. Huselid 1995). As a result, to understand the effects of HRM it is necessary to adopt a holistic view of the overall construction of HR practices that captures the organisation’s human resource (HR) architecture. A HR architecture is unique for each type of organisation (Boxall and Purcell 2008) and can be defined as a set of interrelated components of HRM such as HR policies, principles, or practices (Colbert 2004; Lepak and Snell 1999; Ridder and McCandless 2010) or as the overall internally consistent and coherent HR system structure of an organisation (Arthur and Boyles 2007).

To fill these gaps in research, this mixed methods study investigates the link between two leadership styles and the HR architecture in the educational context. More specifically, this study focuses on the relationship between principals’ instructional and transformational leadership style and the configuration of a bundle of HR practices by primary school principals. In this study we choose to analyse the HR architecture from a school principal’s perspective since principals are seen as street-level human capital managers in education (Milanowski and Kimball 2010; Donaldson 2013) and previous research stressed the important role of principals in the configuration of single HR practices (e.g. Baker and Cooper 2005; Boyd et al. 2011; Papa and Baxter 2008). Finally, this study focuses on the HR architecture for ‘new teachers’ given the growing awareness of the need for particular attention and support to new teachers (TALIS 2008) and the need for more coherent and consistent HR practices for new teachers (e.g. Koppich et al. 2013; Kwan 2009). Three key HR practices that are critical to attract and retain new teachers are central in this study: teacher hiring, induction and awarding tenure to teachers.

## Context of the study

This study focuses on the configuration of HR practices for new teachers in Flemish primary schools (Belgium). In Flanders, as in many other European countries, HR responsibilities are decentralised to the individual school boards (both in primary and secondary education). Regarding HR practices for new teachers, this means school boards are responsible for teacher hiring (recruitment and selection), induction and awarding tenure (European Commission 2013). Yet, in practice, school boards delegate most HR responsibilities related to the management of new teachers to individual schools. In contrast with most secondary schools, in Flemish primary education no multiple management levels exist which means that principals acquire full autonomy to develop and implement HR practices (Devos et al. 2004). Although school boards are officially responsible for HRM, they almost always acquiesce in HR decisions made by the principals (Devos et al. 2004; Devos et al. 2016; Devos et al. 2014). While principals receive large autonomy in HRM, little attention is given to training of principals in HRM. Although, in Flanders a preparation program for principals exists, it is not mandatory. Each principal receives a budget for professional development which can be used for professional development activities. As a result, principals are free to decide to undertake training in HRM. Yet, recent research indicated that beginning principals experience there is a lack of training in HRM skills in existing professional development programs and need more in-service training on HRM (Staelens et al. 2014).

Given this large autonomy and relatively unpreparedness of principals, research shows that HR practices for new teachers are implemented in various ways in Flanders (Devos et al. 2004, 2014, 2016). Flemish principals use, for example, various recruitment procedures such as posting job openings on websites, consulting fellow principals or using a school specific recruitment pool. Moreover, selection procedures vary from a short phone call or e-mail to an in-depth job interview with the principal (and other school team members). Also teacher induction practices are installed in different ways; most of the time they include one or more of the following activities: delivering a school guidebook or a 1-day orientation in the school, a (in)formal assignment of another teacher to act as a mentor, regular feedback moments with the principal (e.g. after classroom observation, during performance appraisal) and/or professional development activities (e.g. a content specific training) (Devos et al. 2004). Finally, although the practice of awarding tenure to new teachers is seldom studied in the Flemish context, research by Devos et al. (2014) shows awarding tenure to new teachers varies from a pro forma decision without any evaluation of the teacher to a thoughtful and shared decision after a clear evaluation process. Yet, in regard to the latter, schools are not obliged to formally evaluate teachers with a temporary appointment within the frame of the first 3 years. The Flemish teacher evaluation policy only obliges schools to formally evaluate all their teachers every 4 years (Department of Education 2009).

Furthermore, it is important to note that the large HR autonomy in Flemish schools does not imply a systematic evaluation or accountability system (Day et al. 2007). Yet, there are specific regulations of the Flemish educational authority related to the appointment of teachers which limit principals’ discretion partially (Devos and Vanderheyden 2002). Once new teachers are hired, their teaching career consists of two key stages. First, new teachers are given a 1-year (or shorter) temporary position. Principals can decide to (dis)continue this temporary position. After three school years (at minimum), teachers can reach the second career stage: tenure. Once tenured, a teacher’s job is secured in every school of the same school network. School networks are groups of schools within a given geographical area. Schools within the same network can make joint decisions in domains such as resources, staff, strategy and education. Joint decisions about awarding tenure to teachers, for example, are important since in case of vacancies, tenured teachers have priority over temporary teachers (i.e. seniority rule) within all schools of the same school network. This means that only if no tenured teachers are available or willing to fill a position within the school network, a new teacher needs to be selected and appointed. In this regard, the tenure decision is an important retention decision for principals.

## Theoretical framework

### The HR architecture for new teachers

While HRM is defined as anything and everything associated with the management of employment relationships in the organisation (Boxall and Purcell 2008), SHRM is focused on management decisions related to policies and practices that shape the employment relationship that are explicitly aimed at achieving individual employee, organisational and/or societal goals (Boselie 2014). Until today, most educational research investigated the effects of and differences in single isolated HR practices such as recruitment and selection, evaluation or induction. Compared to research outside education, few studies investigate the adoption or effects of SHRM in education through the configuration of a bundle interrelated HR practices (Smylie et al. 2004; Smylie and Wenzel 2006). In this regard, recently, a typology of HRM configurations for new teachers in primary education was developed by Vekeman et al. (2015). This typology builds on two orientations: a ‘strategic orientation’ (e.g. Wright and McMahan 1992) and a ‘human resource orientation’ (e.g. Barney 1991). Both orientations are assumed being influential for the configuration of HR practices (Arthur and Boyles 2007; Colbert 2004) and were previously used by Ridder et al. (2012) to develop the architecture of HRM in non-profit organisations.

Informed by strategic HRM perspectives, strategic goals of an organisation define the ‘strategic orientation’. More specifically, an organisation’s HR practices should be designed to fit the organisation’s chosen or emergent goals and each HR practice needs to be aligned with and reinforce the other HR practices. In other words, both a vertical and horizontal fit should be achieved (e.g. Gratton et al. 1999; Ridder et al. 2012). While the ‘vertical fit’ refers to alignment or integration of a bundle of HR practices with organisational goals (Wright and Snell 1998), the ‘horizontal fit’ ensures HR practices pursue similar or complementary goals to reinforce other practices (Kepes and Delery 2007). As in most public sector organisations, in schools goals differ pending on specific values and missions (Bamburg and Andrews 1991) rather than goals being primarily linked to maximizing shareholder value as in for-profit organisations. Moreover, as multiple stakeholders often have heterogeneous interests, conflicting needs and differing views of these organisational values, goals are subject to different interpretations in schools. As a result, compared to for-profit organisations, it can be more difficult to achieve a vertical fit in HRM in schools (Leisink and Boselie 2014). Furthermore, both vertical and horizontal alignment in schools can be complicated by external factors such as resource constraints caused by issues of teacher shortage, high teacher turnover rates, etc. In addition, alignment can be hampered by external demands and rules such as seniority rules, the need to cooperate with other schools, etc. Taken together, we assume that the configuration of a bundle of HR practices for new teachers will vary according to the principal’s strategic orientation. More specifically, a strategic orientated principal is seen as someone who succeeds in aligning school goals with the HR practices (i.e. vertical fit) and in aligning HR practices with each other (i.e. horizontal fit) despite external challenges. This means strategic orientated principals anticipate challenges proactively.

Drawing upon the resource-based view, the second dimension ‘HR orientation’ is based on the assumption that organisation-specific investments are necessary to create value for the organisation (Barney 1991; Wright et al. 2001). It has been shown that this is not only true for profit organisations, but also for non-profit organisations and (semi)-public organisations (e.g. Gould-Williams 2003). In contrast with profit-organisations, school’s HR is decoupled from a market-related logic. Schools adopt a different view on how to invest in HR. Research about new teachers points at a need for support and professional development (Johnson et al. 2001; TALIS 2008). On the one hand, the latter has been linked to attraction and retention of new teachers (e.g. Johnson et al. 2001) and resulting classroom performance (e.g. Desimone et al. 2002). On the other hand, this is linked to new teachers’ job satisfaction (e.g. Shen et al. 2012). In other words, in order to ensure a balanced approach in HRM (Boselie 2014) -in which investments in new teachers create value for both schools and teachers- HR practices could be implemented in such a way that it emphasises support and professional development. In this regard, HR orientated principals implement a bundle of HR practices aiming at enhancing these attributes.

**Fig. 1:**
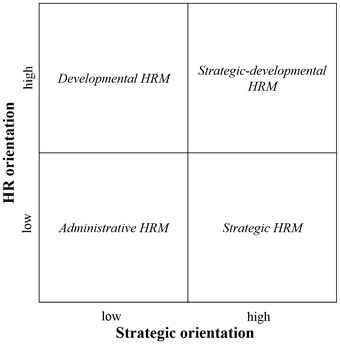
HR architectures in schools

In line with Ridder et al. (2012), we assume that strategic- and HR orientations can range from high to low as the degree of emphasis on each dimension can vary. Juxtaposing these low and high dimensions along the strategic and HR orientations, four quadrants emerge that represent the HR architecture for new teachers in primary education. These four HRM types are depicted in Fig. 1 and summarised in Table 1.

**Table 1:** Differences between HR architectures for new teachers

	Administrative HRM	Developmental HRM	Strategic HRM	Strategic-developmental HRM
*Strategic orientation*	Low	Low	High	High
School goal alignment	Weak vertical or horizontal fit	Weak vertical or horizontal fit	Strong vertical and horizontal fit	Strong vertical and horizontal fit
Coping with external challenges	Reactive approach	Reactive approach	Proactive approach	Proactive approach
*HR orientation*	Low	High	Low	High
Beliefs about human resources	New teachers as resources that need to be acquired	New teachers as resources that need to be developed	New teachers as resources that need to be selected	New teachers as resources that need to be selected and developed

#### Administrative HRM

The administrative HRM is characterised by a weak vertical or horizontal fit. This means practices of hiring, induction and awarding tenure remain isolated from the school goals or do not pursue the same or complementary school goals. In this type, principals approach external demands and challenges reactively. They are aware of external challenges but do not take proactive actions to overcome these challenges. The bundle of HR practices is mainly guided by administrative rules; standard hiring procedures are followed, basic induction practices are installed and the tenure decision is made pro forma. As a result, no real investments are made in new teachers. Teachers are seen as resources that need to be acquired rather than resources that need to be developed.

#### Developmental HRM

The developmental HRM is, as the administrative HRM, characterised by a weak vertical or horizontal fit and a reactive approach towards external challenges. Yet, in this type, the bundle of HR practices is guided by the needs of new teachers rather than administrative rules. Teachers are seen as resources that need to be developed. Principals believe it is important to talk with new teachers, to listen to their needs and to give them time and chances to work on their weaknesses. Yet, principals do not support or guide this developmental process strategically; they do not know towards which school goals they want teachers to develop.

#### Strategic HRM

Principals within the strategic HRM type install HR practices which are vertically and horizontally aligned with the school goals. Moreover, within this type, principals approach external challenges proactively. These principals see advantages in HR practices to respond to these challenges. In contrast to the developmental type, they install induction practices to supervise and assess new teachers’ fit with the school goals as soon as possible. In other words, in the strategic type, new teachers are seen as resources that need to be selected rather than resources that need to be developed.

#### Strategic-developmental HRM

Finally, in the strategic-developmental HRM a balanced focus on both school goals and new teachers’ needs is present. As in the strategic type, HR practices are vertically and horizontally aligned with the school goals and external challenges are approached proactively. Yet, in contrast with the strategic type, teachers are not viewed as good or bad; instead they have a more differentiated perspective of teachers’ strengths and weaknesses. Principals invest heavily in new teachers by supporting their professional development through collaboration and teamwork with other teachers within the school. New teachers are seen as resources which need to be both selected and developed.

### School leadership styles

Although a variety of conceptual models have been employed over the past 25 years of research into educational leadership, two major approaches have predominated: instructional and transformational leadership (Hallinger 2003). In this study we focus on these two leadership styles as both have gained the most support in the educational literature (Leithwood and Jantzi 2006; Robinson et al. 2008). An instructional leadership conceptualisation—drawn from the effective schools literature (e.g. Hallinger and Murphy 1986)—dominated studies from the early to late 1980s. Around 1990 researchers began to shift their attention to leadership models construed as more consistent with evolving trends in educational reform such as empowerment, shared leadership, and organisational learning. The most frequently used model of this variety has been transformational leadership (e.g. Bass 1997; Leithwood and Jantzi 2000). In what follows, both leadership styles are discussed more in detail.

#### Instructional leadership

During the early 1980s several conceptualisations of instructional leadership emerged simultaneously. However, the most frequently used conceptualisation of instructional leadership was developed by Hallinger (2000). This model proposes three dimensions of the instructional leadership construct: (1) defining the school’s mission, (2) managing the instructional program, and (3) promoting a positive school learning climate. Defining the school’s mission includes working with the staff to ensure that the school has clear and measurable goals that are clearly communicated throughout the school community. These goals are primarily concerned with the academic progress of the students. Managing the instructional program requires the school principal to be deeply involved in the school’s curriculum, which includes supervising instruction in the classroom, managing the curriculum, and monitoring students’ progress. The principal also leads improvement of the school’s culture and climate by ensuring there is a high standard of excellence, with high expectations adopted by the school community. This includes promoting professional development, providing incentives for students and staff, maintaining visibility, as well as protecting the time needed for classroom instruction from being consumed by managerial duties.

#### Transformational leadership

During the 1970s and 1980s, transformational leadership was introduced as a theory in the general leadership literature (e.g. Bass 1997; Howell and Avolio 1993). As part of a general reaction against the top-down policy-driven changes that predominated in the 1980s and the directive imagery encompassed in the instructional model, this theory drew the attention of the educational community during the 1990s. Leithwood and his colleagues have carried out the most substantial adaptation of Bass’ transformational leadership construct into the educational field. Leithwood et al. (1998) distinguish seven features of the transformational leadership model: individualised support, vision, shared goals, intellectual stimulation, culture building, rewards, high expectations, modeling. First, this model assumes leadership may well be shared, coming from teachers as well as from the principal (Leithwood and Jantzi 2000; Louis and Marks 1998). Second, the model builds on behavioural components such as individualised support, intellectual stimulation, and personal vision. Leithwood’s conceptual model has been subjected to extended investigation over the past decade which yielded a knowledge base concerning the application of this leadership model in education (e.g. Leithwood and Jantzi 2000). Table 2 summarises the main features and differences of both leadership styles.

**Table 2:** Characteristics and differences instructional and transformational leadership

	Instructional leadership	Transformational leadership
*Characteristics*	Defining the school mission Framing clear school goals Communicating clear school goals Managing the instructional program Supervision and evaluating instruction Coordinating curriculum Monitoring student progress Creating a positive school learning climate Protecting instructional time Promoting professional development Maintain high visibility Providing incentives for teachers Providing incentives for learning	Individualised support Vision Shared goals Intellectual stimulation Culture building Rewards High expectations Modeling
*Differences*	Top-down focus on approach to school improvement First-order target for change Managerial or transactional relationship to staff	Bottom-up focus on approach to school improvement Second-order target for change Transformational relationship to staff

Hallinger (2003) suggests that several criteria may be useful in distinguishing instructional from transformational leadership. First, instructional leadership proposes a more directive and top-down approach, while transformational leadership functions more from the bottom-up. Some scholars characterised instructional leadership as a top-down approach to leadership because of the emphasis on coordination and control. In contrast, transformational leadership is often seen as a type of shared or distributed leadership given the focus on stimulating change through bottom-up participation. A second conceptual distinction contrasts the way by which leadership achieves its effects, through first-order versus second-order changes in the school. Instructional leadership is defined as targeting first-order variables in the change process (e.g. through setting school-wide goals, direct supervision and coordination of the curriculum). In contrast, transformational leaders seek to generate second-order effects by increasing the capacity of others in the school to produce first-order effects on learning (e.g. through articulating the school vision and creating a supportive culture) (Leithwood and Jantzi 2000). A final distinction has evolved around the conceptual dichotomy of transactional versus transformational leadership (e.g. Howell and Avolio 1993). Instructional leadership can be seen as transactional in the sense that the principal manages and rewards school members toward a predetermined set of goals. In contrast, transformational leaders create a common vision, create a consensus among schools members, and inspire followers to accomplish this vision through a more autonomous process (Hallinger 2003). Even though there exists overlap in these two theories of leadership, these differences create a clear distinction between them (Shatzer et al. 2013).

### Linking the HR architecture for new teachers to school leadership styles

While the effects of both instructional and transformational leadership on school effectiveness and both teachers’ and students’ outcomes have been examined (Hallinger and Heck 1998; Leithwood and Sun 2012; Marks and Printy 2003; Robinson et al. 2008; Shatzer et al. 2013), few studies investigate the direct relationship between these leadership styles and the configuration of HR practices in schools. Yet, the existing theory suggests both instructional leadership and transformational leadership include a variety of actions that can be linked to the configuration of HR practices.

First, in order to ensure a *strategic orientation* in the configuration of HR practices, clear school goals are necessary. Although both instructional and transformational leadership have been linked to vision and goals, these constructs are conceptualised differently. The instructional leadership literature asserted that goal-related constructs (e.g. vision, goals) must contain an academic focus (Robinson et al. 2008). In contrast, the application of transformational leadership to education (e.g. Leithwood 1994), left open the ‘value’ question as to the focus of the vision and goals (Hallinger 2011). However, having a clear vision or goals is not enough. To manage human resources strategically, school goals should also be aligned with the different HR practices (vertical fit) and consistency among various HR practices should be ensured (horizontal fit). In order to resource ‘strategically’, Robinson et al. (2008) stated leadership activity should be about securing resources that are aligned with instructional purposes, rather than leadership skill in resourcing per se (Robinson et al. 2008). In the same way, Kimball (2011) stressed it is important in this regard to ensure the teacher competencies necessary to accomplish the school goals are made explicit and are centered on both instructional leadership actions and HR practices. Moreover, he states—in line with Robinson et al. (2008)—instructional leadership typically includes a variety of actions that are tight to a strategic orientation in HRM. In line with Hallinger’s (2003) conceptualisation of instructional leadership, instructional leaders: (1) set clear school goals with an academic focus which are reflected or aligned with the criteria to hire and to tenure new teachers; (2) observe and evaluate teachers’ teaching practices in order to see whether new teachers fulfil the criteria or not and; (3) promote a positive school learning climate through the HR practices they install for new teachers.

Second, in order to ensure a *HR orientation* in the configuration of HR practices, principals should be aware of the development needs of new teachers and should configure HR practices according to these specific needs. Transformational leadership can be linked to a high HR orientation as transformational leaders are characterised by individualised consideration of the needs of their employees. Transformational leaders pay attention to individual and personal differences in needs development and growth and provide necessary resources to help followers to realise their dreams. Transformational leaders listen attentively and pay special attention to followers’ needs for achievement and growth by acting as mentors or coaches and by encouraging followers to take on responsibility in order to develop their potential (Avolio 1999; Bass and Avolio 1994). Although the relationship between HR orientation and transformational leadership has not been explicitly examined in previous research, indirect support for it can be found in studies showing that transformational leadership is associated with so-called human capital enhancing (Zhu et al. 2005) or commitment-oriented HRM (Vermeeren 2014). Human capital enhancing or commitment-oriented HRM seeks to achieve competitive advantage through the strategic development of a highly committed and capable workforce (Huselid 1995). Three factors underpin this kind of approach towards HRM. First, there is a distinctive philosophy which emphasises employee commitment and motivation. Second, relations of trust allow scope and flexibility for employees to exercise influence. Third, culture and leadership styles become important focuses for action in their own right (Zhu et al. 2005).

Taken together, while an instructional leader is concerned with the control and supervision of instruction in order to accomplish the school goals, a transformational leader will focus on teachers’ motivation and commitment and will invest time and effort to stimulate teachers’ professional development towards these school goals (Leithwood 1992). To conclude, based on the existing theory, this study supposes both instructional and transformational leadership will be important to manage new teachers. While instructional leadership is supposed to be related to the strategic orientation of principals, transformational leadership is supposed to be associated with principals’ HR orientation in configuring a bundle of HR practices for new teachers.

## Methods

### Research design

A mixed methods research design is adopted in this study, in which we combine both qualitative and quantitative methods into a single study. More specifically, in this study, we followed a convergent (or concurrent or parallel) mixed methods design (Creswell 2012) in which both qualitative and quantitative data were collected simultaneously, merged and used together. As the purpose of this study is to gain insight in the configuration of a bundle of HR practices for new teachers by principals and its relationship with principals’ leadership style, this particular mixed methods study attempts to answer the following research questions:

1. How do principals configure a bundle of HR practices for new teachers?
2. What is the relationship between principals’ leadership styles and the configuration of HR practices?
  - What is the relationship between principals’ leadership styles and strategic orientation?
  - What is the relationship between principals’ leadership styles and HR orientation?
  - What is the difference in principals’ leadership style between the administrative, developmental, strategic and strategic-developmental HRM type?

First, in order to answer the first research question, qualitative data were collected through semi-structured interviews with school principals. By using a qualitative research method to answer this research question—instead of a quantitative—principals were given the opportunity to talk freely about the visions, beliefs and priorities they have about the management of new teachers in their own school. Moreover, the advantage of the qualitative data is that it offers us many perspectives on the study topic and a complex picture of the situation (Creswell 2012). Following different authors (e.g. Huselid and Becker 1996; Vermeeren 2014), we believe asking the school principal is an adequate measure to get insight in the configuration of HR practices. Furthermore, we asked principals—instead of teachers—to describe the configuration of HR practices in order to rule out common method bias in investigating the relationship between the configuration of HR practices and teachers’ self-reported perceptions of their leader.

Second, in order to answer the second research question, the qualitative data were converted into numerical scores (after qualitative data analysis) and analysed statistically, combined with the quantitative data on school leadership styles (which were collected through a teacher survey). This approach is identified by Onwuegbuzie and Teddlie (2003) as ‘data transformation’ and ‘data correlation’ which represents two stages of their seven-stage conceptualization of mixed methods data analysis process. Data transformation was carried out by converting the qualitative data into numerical codes that can be represented statistically (i.e. quantised data). This was done in order to be able to relate the qualitative (but quantised data) to the quantitative data obtained through the teacher survey (i.e. ‘data correlation’). Quantitative data yield specific numbers that can be statistically analysed, can produce results to assess the frequency and magnitude of trends, and can provide useful information if you need to describe trends about a larger number of people (Creswell 2012). As commonly done in educational research, a survey design was used to measure principals’ leadership styles. More specifically, we asked teachers to rate the leadership style of their principal as both inside and outside the educational research field it is recognised that studying subordinates’ perception of leadership generally provides more accurate ratings than leaders’ self-rating (Atwater and Yammarino 1992). Following the Leader Member Exchange theory, the leadership style of the principal was estimated by aggregating the reported perceptions of the teachers regarding the leader’s style (Rickards 2006).

### Procedure

During the interview with the principal the research purpose was explained and principals were asked to describe how they configure a bundle of HR practices for new teachers in their school. Next, principals were asked to send an electronic link for the online survey to all teachers with a teaching assignment in the school. A minimum teaching experience of 3 months in the school was set since a minimum period of time is necessary for teachers to ensure that they had adequate opportunities to observe and accurately rate their principal’s behaviour. The principal had no access to the answers on the survey and teachers’ responses were completely anonymous. All respondents gave informed consent in which the study proposes, procedures, and the methods in place to protect the anonymity of all respondents were explained in a detailed way.

### Qualitative data collection and analysis

#### Sample

To select the schools we used a stratified random sampling. In Flanders, schools are grouped in educational networks according to their governance structure. In total, 14 (19 %) publicly financed schools run by the Flemish authority, 20 (26 %) publicly financed schools run by the municipalities and 41 (55 %) publicly financed schools but privately run participated in this research. This division mirrors approximately the proportion of each school educational network in the population of Flemish primary schools. In each school the principals was interviewed. The principal sample included 45 % male and 55 % female principals. Principals in the sample were between 29 and 59 years old with an average of 48 years. The amount of years that they were appointed as principal in their school fluctuated between one and 24 years, with an average of 8 years. In total 24 were temporary assigned and 51 were tenured as principal in the school.

#### Instruments

Semi-structured open-ended (face-to-face) interviews were used to identify how principals configure a bundle of HR practices for new teachers. The open-ended questions treated themes such as HR-procedures for hiring, induction and awarding tenure, barriers or constraints to install HR practices, reasons for the configuration of HR practices, school goals, etc. (see Appendix for the interview protocol). The interviews lasted on average 60 min and were recorded and transcribed verbatim.

#### Analysis

To analyze the interview transcripts, different steps were taken. First, thematic summaries were created after completing each interview in order to reduce the data (Miles and Huberman 1994). These summaries included broad categories (e.g. hiring, induction, tenure, etc.) with subcategories (e.g. hiring criteria, hiring tools, hiring constraints, solutions to overcome constraints, etc.). Second, the interviews were coded inductively in these descriptive categories. Third, deductive coding was used based on the dimensions of strategic orientation and HR orientation. Categories for each of the two dimensions were used which were developed in a study by Vekeman et al. (2015). Along the strategic orientation dimension, the analytic categories included: ‘school goal alignment’ and ‘coping with external challenges’. The category ‘school goal alignment’ reflects the vertical fit (i.e. the alignment between the school goals and HR practices) and the horizontal fit (i.e. the degree that HR practices pursue the same or complementary school goals). The category ‘coping with external challenges’ reflects the way principals approach external challenges. The principals were scored low/high on the strategic orientation when respectively: (1) a weak/strong vertical or/and horizontal fit was noticed and (2) a reactive/proactive approach is taken towards external challenges. The HR orientation of principals was analysed looking at the extent to which the principal considers the development needs of their new teachers in the application of HRM. The principals were scored low/high on the HR orientation when respectively teachers are seen as resources that need to be acquired or selected/be developed. Based on these categories, within-case analysis was conducted and all 75 schools were classified according to two possible strategic orientations (low or high) and two possible HR orientations (low or high). Finally, for both orientations the qualitative scores were transformed in numerical scores: ‘low’ was quantised as 0 and ‘high’ was quantised as 1.

Procedures helped increasing the validity: peer review and debriefing (Creswell and Miller 2000). Also, considerable time was spent (re)reading the transcripts (Patton 1980). Finally, a researcher not familiar with the study coded—after training—independently the data for both the strategic and HR orientations. Ten interviews were double-coded. Coding differences were analysed and discussed and resolved by returning to the interview transcripts and specific codes (Miles and Huberman 1994).

### Quantitative data collection and analysis

#### Sample

After deleting respondents with missing values, the responses of 1058 teachers were used in this study. This sample has an average response rate of 70.43 % in the participating schools. A response rate around 70 % is generally recommended as acceptable (Johnson and Christensen 2008). However, this recommendation is based on the assumption that respondents and non-respondents are fairly similar. Comparing different teacher characteristics of our sample with the whole Flemish teaching population, we can state this assumption is not violated in our study. The teacher sample included 12 % male and 88 % female teachers. Teachers’ age ranged from 20 to 64 years, with an average of 38 years. The average experience in their current school was 12.5 years, varying from less than 1 year to 39 years. These descriptive measures reflect the male–female, age and experience distribution in Flemish primary school teaching population.

#### Instruments

Each principal’s leadership style was measured by a teacher survey in which existing leadership scales were used. Instructional leadership was measured through a scale of Louis et al. (2010). The scale contains items such as: ‘My principal clearly defines standards for instructional practices’ or ‘How often this school year has your principal given you specific ideas for how to improve your instruction?’. Two items from the original scale were deleted due to the specific context of primary education. All items on both scales were scored by teachers on a range from 1 (never) to 5 (always). For transformational leadership a scale by Hulpia et al. (2009) was used. This scale is used and validated in the educational context and is based on strength of vision (De Maeyer et al. 2007), supportive behavior (Hoy and Tarter 1997), providing instructional support, and providing intellectual stimulation (Leithwood and Jantzi 1999). Exemplary items are ‘My principal helps teachers’ and ‘My principal encourages me to pursue my own goals for professional learning’. All items were scores by teachers on a range from 1 (strongly disagree) to 5 (strongly agree).

#### Analysis

First, to ensure construct validity of the scales, factor analyses and item analyses were performed. An exploratory factor analysis (with promax rotation) was performed in order to check whether both leadership styles were underlying the items. Based on this factor analysis, one item from the instructional leadership scale was deleted due to cross loadings. Next, reliability analyses were conducted for the scales which showed good Cronbach’s alpha values of 0.88 for the instructional leadership scale and 0.94 for the transformational leadership scale.

Second, the teachers’ scores on the leadership scales were aggregated at the school level. The interclass correlation ICC(2) (Bliese 2000) was calculated to determine the magnitude of agreement using mean squares from a one-way ANOVA (formula: between mean square–within mean square/between mean square). For instructional leadership the ICC(2) was 0.88 and for transformational leadership the ICC(2) was 0.87, indicating aggregation was justified (Glick 1985). As the principal’s leadership style is viewed homogeneously in this study—according to Antonakis et al. (2004)—it is justifiable to aggregate the individual data to the group level.

Third, binary logistic regressions were employed to examine what the relationship is between principal’s leadership style and the configuration of a bundle of HR practices. The instructional and transformational leadership variables were the independent variables, ‘strategic orientation’ and ‘HR orientation’ were the separate dependent variables. A Cook’s distance analysis was performed to identify outliers based on having a Cook’s Distance value of greater than 1.0 (Hosmer and Lemeshow 2000). In the strategic-developmental HRM type, one outlier with a Cook’s distance value of 1.064 was detected. This case was removed for all analyses in this study. In order to examine differences in leadership style between the four HRM types, one-way ANOVA tests were used followed with appropriately conservative post hoc tests in relation to homogeneity of variance.

## Results

As noted earlier, qualitative interviews with principals provided rich information about the way they configure a bundle of HR practices for new teachers in their school (i.e. HR architecture). Following the presentation of the qualitative results, quantitative results extend the qualitative analyses which examine the link between the HR architecture and principals’ leadership style. As qualitative data were analysed first and were transformed into quantised data in order to link with the survey data, qualitative results are presented first below, followed by the quantitative findings.

### Qualitative results: How do principals configure a bundle of HR practices for new teachers?

#### Administrative HRM

In total 38 principals were classified in the administrative HRM type. First, this type is characterised by a weak vertical or horizontal fit. In cases where there is a weak vertical fit, HR practices remain completely isolated from the school goals. One principal indicated, for example, that ‘openness to every child’ is the central school vision. However, no specific school goals are formulated towards that vision. Consequently, no clear hiring or tenure criteria are used which reflect that vision. In the cases where there is a weak horizontal fit, principals align some of the HR practices with the school goals while not all HR practices pursue the same or complementary goals. Some principals, for example, align their tenure criteria or hiring criteria with the school goals while the other practices are not configured strategically. Furthermore, the interviews with principals classified in this HRM type suggested the tenure decision is not based on a real evaluation of new teachers. Some principals make the tenure decision mainly pro forma after 3 years, as one principal stated:

We have some standard criteria, but actually all teachers get into the tenure-track position automatically. Teacher tenure is a teacher’s right, you know. We cannot take away this right from teachers.

In the administrative HRM type a reactive approach is taken towards external challenges such as limited supply of skilled teachers, seniority rules which limit a proactive hiring planning, a short period before the tenure-track decision, limited resources to support new teachers, etc. Actually, the interviews showed that HRM does not play a role in coping with these challenges. For example, most principals are aware of the limited supply of skilled teachers but do not invest in recruiting and attracting skilled teachers proactively. One principal said: ‘It is very hard to find a good teacher. Sometimes I hire a teacher and know it isn’t a good teacher but I can only work with what I get, isn’t it?’. In line with this, principals perceive new teachers as resources that need to be acquired rather than resources that need to be developed or selected strategically. Principals do not invest systematically in new teachers that go beyond short-term necessities of basic orientation and induction. Some of the principals referred to a lack of time and resources to explain why only basic induction practices were installed or explicitly said new teachers do not need a lot of support, as the following quote illustrates:

Support for new teachers? On long term basis, after four years, we have the mandatory teacher evaluations. On short time basis, at the first day, we sign the necessary papers in order that teachers get there salary. And, than I say also: If there is something, just ask your colleague or ask me if necessary. But actually, they don’t need a lot of support and guidance.

Taken together, principals’ configuration of HR practices within the administrative type is characterised by a focus on bureaucratic rules rather than a focus on own school goals or needs of new teachers. During the interview, principals referred to standard procedures to recruit new teachers, to standard documents which new teachers get with the necessary information about the school regulations, to the necessary paper work that needs to be filled out for a tenure-track position, etc.

#### Developmental HRM

Twenty-one principals were classified in the developmental HRM type. As in the administrative HRM type, principals do not operationalise school goals sufficiently to direct HR practices for new teachers or do not align all HR practices with the same or complementary school goals (no vertical or horizontal fit). One principal said, for example, that the school vision is ‘caring for all kind of pupils’, without mentioning aligned school goals. As a result, in most cases the school vision is not reflected in the configuration of HR practices. Principals do not hire and retain teachers based on clear criteria. Actually, most principals indicated it is difficult to say what they are looking for. Principals do not use sophisticated HR procedures and do not plan the hiring and tenure decision proactively. Furthermore, principals referred during the interview to the same external challenges as principals in the administrative HRM. Principals within the developmental HRM type also tent to cope with external challenges using a reactive approach. They do not take proactive HRM decisions (e.g. recruiting teachers proactively; making proactive tenure decisions). The following quote illustrate this approach:

It is difficult for me to make the decision to keep a teacher or not after two or three years. And I don’t want to make that hard decision for new teachers … We should give teachers more time to develop, to feel connected with the school … You don’t see that immediately, not at the time you hire them, nor at the time we are forced to make a tenure decision.

Yet, in contrast with principals in the administrative HRM type, the interviews showed that principals see new teachers as resources that need to be developed rather than resources that need to be acquired. In order to do that principals believe it is important to talk with new teachers, to listen to their needs, to support them and to give them the chance to work on their weaknesses. The following quote illustrates this:

I’m not the person that deselects teachers who work only one or two years in my school. I think teachers should get some chance to develop. Actually, I often argue about this with other principals. Some principals tend to deselect teachers very soon because they fear ‘bad’ teachers get tenured. I don’t agree with that. I think you need to give teachers the time to develop and give them the chance to make some mistakes.

Taken together, HRM in the developmental type is characterised by a focus on the internal needs of new teachers rather than administrative procedures. Principals recognise new teachers have different needs and belief HRM should be orientated towards new teachers’ needs. In this regard, new teachers are supported to develop professionally according to their own needs rather than according to the goals of the school.

#### Strategic HRM

Only seven principals were identified as high in their strategic orientation and low in their HR orientation. Principals, within this HRM type, all set clear school goals and ensure both that HRM is aligned with the school goals (i.e. vertical fit) and that single HR practices pursue the same or complementary school goals (i.e. horizontal fit). As a result, principals in this HRM type set clear and strategic hiring criteria, plan the recruitment of new teachers proactively, and developed sophisticated hiring procedures to screen and select new teachers. The same is true for the tenure decision which is proactive, based on clear and strategic criteria and is made after various informal and formal evaluations of teachers’ practice. One principal explained:

The hiring and tenure decision is extremely important for me and I take a lot of time for it. First, I read CV’s, I contact other schools they worked in or did their internship, … […] After this screening, I interview some candidates profoundly. I ask them a whole set of questions and ask them to solve some problems which already occurred within our school to see whether he/she would be able to attain what we stand for in our school.[…] After they are hired, we follow a strict evaluation policy. After the first weeks I have a conversation with them. I force myself to visit new teachers twice a year and to discuss their performances afterwards. Based on this information, but also on informal contacts, I’m able to evaluate teachers’ quality and teachers’ fit with our school vision after one year. They need to fit, otherwise I will discontinue the contract. It’s hard, I know, but I have a big responsibility. I need to ensure my pupils get good education.

Furthermore, these principals cope with external challenges using a strong proactive approach. They all seem to be aware of the external challenges they are faced with and see advantages in HRM to respond to these challenges. One principal said for instance: ‘Yes, there is shortage of good teachers but we know that in advance. We need to search teachers proactively and set clear criteria if we want to find them’. As a result, hiring, induction and tenure practices are installed strategically. Moreover, principals indicated it is important to install various informal and formal activities (e.g. classroom observations, performance appraisal conversations, mentoring) for new teachers. In contrast to the developmental HRM type, where principals install induction practices to support and develop new teachers, principals indicated these practices are necessary to supervise and assess new teachers’ fit with the school goals as soon as possible. According to them such an approach is necessary given the external challenges such as the ‘short period before the tenure-track position’ and ‘the immunity of the tenure-track position’. Actually, in this HRM type, the focus on selective and proactive staffing takes clearly precedence over giving teachers time to develop. In other words, in the strategic HR type new teachers are seen as resources that need to be selected rather than resources that need to be developed, as one principal said:

If you notice teachers do not function as you expect them to do, it is important to pull them out as soon as possible—before they get enlaced in your team. It has only disadvantages if you keep on trying it with that person without result. If you, as a principal, do not know if it is a good teacher after one or 2 years … than the question is: “When do you know?”. You need to invest time. As a principal, you need to take time to select the best possible candidates and to supervise new teachers as much as possible in the first year of their appointment. This is necessary to make a deliberate retention decision as soon as possible.

To sum up, within the strategic HRM ype, the management of new teachers is characterised by a focus on school goals, rather than a focus on new teachers’ development needs or administrative rules. In order to accomplish school goals through the configuration of HR practices, principals believe new teachers should be selected strategically, rather than developed.

#### Strategic-developmental HRM

Finally, nine principals were identified as both high strategic orientated and high HR orientated. Principals set clear school goals, align school goals with the different HR practices and ensure that all HR practices pursue the same or complementary goals (i.e. both vertical and horizontal fit). Actually, principals within this HRM type are strategic in hiring and retaining teachers. Therefore principals have clear and strategic hiring criteria, use sophisticated hiring procedures and plan the hiring decision proactively. The same is true for the tenure decision. Principals know very well which kind of teachers they want to retain. The strong integration between goals and HRM can be illustrated by the following quote from a principal:

Our vision? It is extremely important here … We constantly question ourselves in order to become better for our school, our teachers and our pupils. What is it what we do and why? ‘Why’ is a sacred word here … In our school, breeding or nurturing pupils is central. We are at service of breeding pupils but it is not a goal on its own. Learning is at service of breeding. Moreover, we do not follow a closed model. We need human interpretation in every situation. Education is not the same in every school and also not for every child. Therefore we need to be critical all the time and try to feel that what we do is in line with what pupils, parents and we as members of the school stand for. We hire and retain only authentic teachers, teachers who fit within this picture; preferably teachers as unique as possible, yet those who share our common school goals and vision.

Moreover, as in the strategic HRM type, principals adopt a proactive approach towards external challenges. Rather than succumbing to external challenges, these principals try to search for possible solutions in their HRM to overcome these challenges. Furthermore, new teachers are seen as resources which need to be both selected and developed. In contrast to the strategic HRM, teachers are not viewed as good or bad; instead they have a more differentiated perspective of teachers’ strengths and weaknesses. One principal explained:

I need to be selective. I need to select and retain only those who fit here. I need to do this because they need to work intensively together with others in our school. So, they have to fit in and need to share the same ideas. However, this does not mean I deselect teachers immediately who seem not to fit. You need to give teachers time to develop and expand themselves, to become part of the team, to internalise our school vision, … But it is naive to believe new teachers can do this all by their own. As a school, as the entire school team, you need to support teachers.

Taken together, principals within the strategic-developmental HRM are characterised by a balanced focus both on school goals and internal needs of new teachers. They believe school goals can be accomplished through HRM that focus on selection and development of new teachers.

### Quantitative results: What is the relationship between principals’ leadership styles and the configuration of HR practices?

#### Descriptive results

Means and standard deviations for the leadership variables are presented in Table 3. First, these descriptive results reveal that principals are perceived more as transformational leaders (M total = 3.95) than instructional leaders (M total = 3.01) by their teachers. Second, these descriptive results suggest that the leadership style slightly differs according to both the strategic and HR orientation and the HRM type. Teachers’ average leadership perceptions for both instructional and transformational leadership in the administrative and developmental HRM type center around the total mean for instructional and transformational leadership. In the strategic and strategic-developmental HRM types, teachers’ average leadership perceptions for both instructional and transformational leadership deviate from the total mean score. The developmental HRM type showed the smallest average score on the instructional leadership (M = 2.81) and transformational leadership (M = 3.85), in contrast with the strategic HRM type showing the highest average score on instructional leadership (M = 3.75) and transformational leadership (M = 4.32).

**Table 3:** Means and standard deviations of study variables

	N	Instructional leadership	Transformational leadership
		M (*SD*) Min 1–Max 5	M (*SD*) Min 1–Max 5
Strategic orientation			
Low	59	2.88 (*0.47*)	3.86 (*0.46*)
High	15^a^	3.51 (*0.46*)	4.32 (*0.16*)
HR orientation			
Low	45	3.05 (*0.53*)	3.94 (*0.44*)
High	29^a^	2.95 (*0.52*)	3.98 (*0.50*)
HR architecture			
Administrative HRM	38	2.92 (*0.43*)	3.87 (*0.43*)
Developmental HRM	21	2.81 (*0.53*)	3.85 (*0.52*)
Strategic HRM	7	3.75 (*0.52*)	4.32 (*0.19*)
Strategic-developmental HRM	8^a^	3.30 (*0.28*)	4.32 (*0.14*)
Total	74^a^	3.01 (*0.53*)	3.95 (*0.46*)

^a^In the strategic-developmental HR architecture, one outlier was detected and removed for all analyses in this study and therefore not reported in the table (see results)

#### Logistic regression analysis

##### Relation between leadership style and strategic orientation

To answer research question 2a, a binary logistic regression was performed including instructional and transformational leadership as the independent variables and the strategic orientation of principals as the dependent variable. Table 4 summarises the results of the regression model. The logistic regression model was statistically significant, *χ* ^2^ (2) = 26.631, *p* = 0.000. The model explained 47.6 % (Nagelkerke *R* ^2^) of the variance in strategic orientation and correctly classified 83.8 % of cases. Both instructional leadership [Wald(1) = 6.914, *p* = 0.009] and transformational leadership [Wald(1) = 4.290, *p* = 0.038] added significantly to the model. More specifically, the results ratio shows that principals who are perceived more as instructional leaders or transformational leaders are more likely to be in the group of high strategic orientated principals, rather than the group of low strategic orientated principals. The odds ratio tells us that as principals’ instructional leadership score or transformational leadership score increases with one-unit, the odds of being a high strategic orientated principal is respectively 15.426 times and 22.508 more higher than a low strategic orientated principal.

**Table 4:** Predicting strategic orientation

Variable	*B*	*SE*	Wald	*df*	*p*	Exp (B)
Constant	−23.045	6.839	11.356	1	0.001	0.000
Instructional leadership	2.736	1.041	6.914	1	0.009	15.426
Transformational leadership	3.114	1.503	4.290	1	0.038	22.508

*B* = regression coefficient; *SE* = standard error; Wald = Wald statistic; Exp (B) = odds ratio. Model *χ* ^2^ (2) = 26.631, *p* < 0.001; −2 log likelihood = 47.979; Cox and Snell *R* ^2^ = 0.302; Nagelkerke *R* ^2^ = 0.476

##### Relation between leadership style and HR orientation

In order to answer research question 2b, a binary logistic regression was performed with the HR orientation as dependent variable and both leadership styles as independent variables. As is shown in Table 5, no significant relationship was found between both leadership styles and principals’ HR orientation [*χ* ^2^(2) = 1.520, *p* = 0.468].

**Table 5:** Predicting HR orientation

Variable	*B*	*SE*	Wald	*df*	*p*	Exp (B)
Constant	−0.820	2.157	0.144	1	0.704	0.441
Instructional leadership	−0.653	0.558	1.366	1	0.242	0.521
Transformational leadership	0.590	0.642	0.847	1	0.357	1.805

*B* = regression coefficient; *SE* = standard error; Wald = Wald statistic; Exp (B) = odds ratio. Model *χ* ^2^ (2) = 1.520, *p* > 0.05; −2 log likelihood = 97.579; Cox and Snell *R* ^2^ = 0.020; Nagelkerke *R* ^2^ = 0.028

#### ANOVA analysis

To answer research question 2c, a one-way ANOVA test was performed. The test’s results showed there are differences in instructional leadership [F(3, 70) = 8.929, *p* = 0.000] between the four HRM types. Bonferroni post hoc tests were used to examine differences in instructional leadership scores between the four HRM types. The results showed that the mean scores for instructional leadership in the strategic HRM type were only significantly higher than the mean scores in the administrative and developmental HRM type. No differences were found between the mean scores for instructional leadership in the strategic and strategic-developmental type. For transformational leadership, the Levene’s test of homogeneity indicated that the assumption of homogeneity of variances was violated. Therefore, the more robust Welch’s ANOVA was utilised. The test showed there are significant differences in transformational leadership between the four HRM types [Welch(3, 24.856) = 12.441, *p* = 0.000]. Since equal variances were not assumed for transformational leadership [Levene Statistic(3, 70) = 4.100, *p* = 0.010], Games–Howell post hoc tests were utilised. The results showed that the mean scores for transformational leadership in the strategic HRM type and strategic-developmental HRM type were significantly higher than the mean scores in the administrative and developmental HRM type. No differences were found between the administrative and developmental HRM type or between the strategic and strategic-developmental HRM type. The results of both post hoc ANOVA tests are summarised in Table 6.

**Table 6:** Results post hoc ANOVA test

	*p* value
*Instructional leadership*	
Administrative	
Developmental	1.000^a^
Strategic	0.000^a^
Strategic-developmental	0.221^a^
Developmental	
Strategic	0.000^a^
Strategic-developmental	0.080^a^
Strategic	
Strategic-developmental	0.362^a^
*Transformational leadership*	
Administrative	
Developmental	0.998^b^
Strategic	0.001^b^
Strategic-developmental	0.000^b^
Developmental	
Strategic	0.008^b^
Strategic-developmental	0.004^b^
Strategic	
Strategic-developmental	1.000^b^

^a^Bonferroni post hoc test

^b^Games–Howell post hoc test

## Discussion

This mixed methods study showed, first, only a minority of principals configure a bundle of HR practices for new teachers strategically. While the idea grows that also in education a strategic approach in HRM is necessary (Davies 2003; Leisink and Boselie 2014; Smylie et al. 2004), the interviews showed that the majority of principals do not align their school goals with the bundle of HR practices for new teachers and do not align HR practices with each other. Moreover, it was striking to notice that almost half of the principals in this study could be classified in the administrative type. Principals within this type are mainly orientated towards bureaucratic rules rather than towards the needs of new teachers or school goals. Taken together, a lot of principals are still concerned with operational procedures of HR practices, rather than ensuring that the bundle of HR practices reflects the strategic goals of their school. Furthermore, the interviews showed there is a disharmony in the way new teachers are managed. Half of the principals in this study seem to recognise new teachers’ development needs. The other half of the principals seem to perceive new teachers as resources that need to be acquired or selected in the first place, rather than resources that need to be developed. This result confirms earlier research showing that principals have different beliefs about the management of new teachers. For example, Youngs (2007) found differences in principals’ awareness of new teachers’ need for support, understanding of induction and convictions about professional development. These beliefs shape -together with their beliefs about leadership, their professional backgrounds and their responses to policy- the way principals undertake efforts to support new teachers (Youngs 2007).

Second, this study showed, that the leadership style of primary school principals is reflected in the way they configure a bundle of HR practices for new teachers in their own school. We found evidence for the relationship between principals’ instructional and transformational leadership style and principals’ strategic orientation in the configuration of HR practices. The more principals are perceived as instructional leaders and transformational leaders, the more likely they are to configure the bundle of HR practices for new teachers high strategically (i.e. HR practices are vertically and horizontally aligned with the school goals). Based on the existing literature, we expected that instructional leadership (rather than transformational leadership) would be linked to the strategic orientation of principals (Milanowski and Kimball 2010; Robinson et al. 2008). Yet, this study points at the importance of both instructional and transformational leadership in configuring a bundle of HR practices high strategically. It seems that the emphasis on articulating and accomplishing school goals—which is characteristic for both leadership styles (Hallinger 2003)—is clearly reflected in the strategic orientation of principals. Moreover, this study indicated that a principal’s HR orientation is not reflected in his/her leadership style. Principals were not perceived as more instructional leaders or transformational leaders if they were identified as high HR orientated instead of low HR orientated. In this regard, our study did not confirm our theoretical expectation that a high HR orientation is reflected in principals’ transformational leadership. Further research is necessary to investigate whether the HR orientation of principals is linked to other factors which are not expressed in their transformational leadership style (e.g. personal beliefs and values).

Third, this study found differences in instructional and transformational leadership between the four HRM types. The mean scores for instructional leadership were significantly higher in the strategic type compared with the administrative and developmental type. Yet, no difference was found between the strategic and strategic-developmental type for instructional leadership. The same was true for transformational leadership: in the strategic HRM type principals’ transformational leadership style was scored as high as principals in the strategic-developmental HRM type. This was surprising as we expected that principals’ transformational leadership score in the strategic-developmental HRM type would be higher than in the strategic HRM type. We expected this because principals in the strategic-developmental HRM type focus on developing new teachers rather than selecting new teachers. In this regard, further research is necessary to clarify why principals in the strategic HRM type are also perceived as strong transformational leaders. Do they show transformational leadership behaviours because they work with a strategically selected pool of teachers which are better to motivate? Or is it because these teachers value this kind of leadership style? And if so, why do they value it? A qualitative design could be used to answer these questions, for example using teacher interviews or observations of principals’ work. Moreover, we found that principals in the strategic HRM type also scored high on instructional leadership. Although there was no significant difference in instructional leadership between the strategic and strategic-developmental HRM type, the mean scores for instructional leadership were the highest in the strategic HRM type and were significantly different from the mean scores in the administrative and developmental HRM type. Principals in the strategic HRM type are characterised by their focus on selecting only those teachers who fit the school goals. It is possible that because they are very selective at the time of hiring and awarding tenure to teachers compared with principals in the other HRM types, they are perceived as more directive and active in their instructional leadership.

## Conclusions

Taken together, this study suggests both instructional and transformational leadership is important to manage new teachers strategically. This result reconfirms the importance of an integrated leadership style in education (Marks and Printy 2003) and contributes to the leadership and HRM theoretical literature in several ways. First, although many studies have been done on educational leadership and single HR practices in education, the present research is the first attempt to integrate the two separate streams of research. Second, this study contributes to the existing literature by adopting a holistic view to look at HRM in education, rather than analyzing isolated HR practices. Finally, while broad agreement exists on the importance of instructional leadership, there is less consensus on what instructional leadership actually is. The traditional instructional leadership literature construes instructional leadership as synonymous with classroom observations and direct teaching of students and teachers. In line with Horng and Loeb (2010) this study shows a different view of instructional leadership is necessary, one that includes strategic HRM as central to instructional improvement.

### Recommendations for further research

While this research contributes to the leadership and HRM literature, more research is recommended in this area to fully understand the relationship between leadership styles and HRM in education. One interesting direction for future research would be to examine other attributes of principals, besides the leadership style, that might affect the configuration of HR practices. In this respect, previous research—outside the educational field—has suggested to examine differences in values or experiences of managers (Gilbert et al. 2011) and managers’ willingness, capacity and competence to implement HRM (Nehles et al. 2006). On the other hand, principals’ HRM and/or leadership style might also be influenced by factors that are not under the control of principals such as characteristics of the teacher population within the school (e.g. teacher quality within the school, teacher demographics, teachers’ need for development,…), school characteristics (e.g. pupil population, school district influence) or labour market features (e.g. demand- and supply of teachers within specific region). Furthermore, it would be interesting to see how teachers perceive HRM in their school and how perceived HRM and actual HRM (i.e. HRM implemented by principals) is associated with teachers’ outcomes such as job satisfaction, organisational commitment or intention to leave. Finally, as this study suggests that principals in the strategic HRM type integrate both leadership styles, the question might be: Should every principal implement strategic HRM for new teachers? Or is strategic-developmental HRM preferable? Is there actually one ‘ideal’ HRM type? To answer these various questions, we believe further research is inevitable which also takes into account teacher outcomes. Future studies are necessary to link the HRM types to teacher outcomes such as teachers’ job satisfaction, organisational commitment and intention to leave the job. Moreover, it would be interesting to investigate whether differences in the configuration of HR practices for new teachers influence the extent to which teachers’ own values ‘fit’ the school values or goals (i.e. Person-Organisation Fit).

### Limitations

As always in research, this study has limitations and needs follow-up in other studies. First, a larger sample of schools would allow us to include more variables (e.g. control variables). Since the preferred ratio of valid cases to independent variables for logistic regression is 20 to 1, only a limited number of variables could be included in this study. In this regard, future research should use a larger sample to test the findings of this study. A second limitation is that we only interviewed principals to gain insight in the HR architecture for new teachers. Although we made use of multiple data sources (i.e. teachers and principals) in order to reduce common method bias, we believe that interviews with teachers and actors at the meso-level and/or direct observation of principals might offer further important information to measure the existent HR architecture. The latter is interesting because what principals say, practice or, apply can be discrepant from what teachers (or other actors) experience (Wright and Nishii 2007). A third methodological limitation that should be taken into account is the cross sectional nature of this study which does not allow to confirm the suggested causality between the principal’s leadership style and configuration of HR practices. More longitudinal research is necessary to study this relationship. A final limitation is that our sample was limited to Flemish primary schools. It would be useful to involve samples from different educational levels since previous research showed HR practices are different (Devos et al. 2004) and leadership effects are stronger at the elementary than secondary school level (Louis et al. 2010). Moreover, this study was carried out in Flanders. It is possible that the specific educational context of Flemish education influences the way principals configure their HR practices. Therefore, it is important to verify the study results in other national or regional contexts.

## Appendix

### Interview protocol

1. Tell me about your recruitment and hiring approach.
  - How does the recruitment and hiring process look like? Which steps do you take, who is involved, which procedures do you follow, etc.?
  - What is the timeline?
  - What are important criteria for you to hire teachers? Which criteria are most important for you?
  - Based on what kind of information you make a hiring decision?
  - What helps you in hiring good teachers?
  - What has influenced the way you approach recruitment and hiring?
  - What barriers do you encounter in teacher recruitment and hiring?
  - Do you need to search for new teachers a lot? How many vacancies do you have each year? What is the reason you need to search for new teachers?
  - Is it hard to recruit teachers? Is it hard to find suitable teachers for the job and your organisation?
  - How do you manage to overcome problems related to the recruitment of teachers?
  - How do you wish you could hire teachers? What is good hiring according to you?
2. How do you induct new teachers within your school?
  - Do you have a specific program for teacher induction?
  - What is your role in induction and who else is involved?
  - What inhibits your involvement? What promotes it?
  - How important do you think is induction for new teachers?
  - What has influenced your approach in induction?
  - How do you wish you could induct teachers? What is good induction according to you?
3. Tell me about your approach towards teacher tenure.
  - How do you make a tenure decision? Based on which information do you make a tenure decision?
  - What are for you important criteria to make a tenure decision?
  - When do you make this decision?
  - How important is the tenure decision for you?
  - What constraints you in making the tenure decision?
  - What helps you in making a good tenure decision?
  - How do you wish you could tenure teachers?
4. Teacher turnover is an important issue in education today.
  - Is there a lot of turnover in your school?
  - What are reasons for the big/little amount of turnover in your schools?
  - Can you control turnover and how do you control it?
  - Which kind of teachers turn over most of the time?
5. Human resource management
  - How, if at all, are the tasks of recruitment, hiring, induction and tenuring related to each other, in your view? In other words, does the way you conduct hiring affect how you approach induction or awarding tenure to teachers?
  - What are priorities for you in the management of new teachers?
  - Are you satisfied with the way you manage new teachers in the school?
6. Can you tell me about the vision of your school?
  - What is the central vision of this school?
  - What are important school goals your school is striving for?
  - Which goals are most important in this school?
  - How important is it for you that teachers accomplish these goals?
  - How important is teacher collaboration in this school? How important is it for you? How important is it for teachers in your school?
  - How important are innovations in this school? How important is it for you? How important is it for teachers in your school?
  - How important is academic achievement of pupils important in this school? How important is it for you? How important is it for teachers in your school?
  - How important is a focus on discipline in this school? How important is it for you? How important is it for teachers in your school?

## Abbreviations

HR: human resource
HRM: human resource management
SHRM: strategic human resource management

## References

[CR1] Antonakis J, Schriesheim CA, Donovan JA, Gopalakrishna-Pillai K, Pellegrini EK, Rossomme JL, Antonakis J, Cianciolo AT, Sternberg RJ (2004). Methods for studying leadership. The nature of leadership.

[CR2] Arthur JB (1994). Effects of human resource systems on manufacturing performance and turnover. Acad Manag J.

[CR3] Arthur JB, Boyles T (2007). Validating the human resource system structure: a level-based strategic HRM approach. Hum Resour Manag Rev.

[CR4] Atwater LE, Yammarino FJ (1992). Does self-other agreement on leadership perceptions moderate the validity of leadership and performance predictions?. Pers Psychol.

[CR5] Avolio BJ (1999). Full leadership development.

[CR6] Baker BD, Cooper BS (2005). Do principals with stronger academic backgrounds hire better teachers? Policy implications for improving high-poverty schools. Educ Admin Q.

[CR7] Bamburg JD, Andrews RL (1991). School goals, principals, and achievement. Sch Eff Sch Improv.

[CR8] Barney JB (1991). Firm resources and sustained competitive advantage. J Manag.

[CR9] Bass BM (1990). From transactional to transformational leadership: learning to share the vision. Organ Dyn.

[CR10] Bass B (1997). Does the transactional–transformational leadership paradigm transcend organizational and national boundaries?. Am Psychol.

[CR11] Bass BM, Avolio BJ (1994). Improving organizational effectiveness through transformational leadership.

[CR12] Beteille T, Kalogrides D, Loeb S (2012). Stepping stones: principal career paths and school outcomes. Soc Res.

[CR13] Bliese PD, Klein KJ, Kozlowski S (2000). Within-group agreement, non-independence, and reliability: implications for data aggregation and analysis. Multilevel theory, research, and methods in organizations.

[CR14] Boselie P (2014). Strategic human resource management—a balanced approach.

[CR15] Boxall P, Purcell J (2008). Strategy and human resource management.

[CR16] Boyd DJ, Grossman PL, Ing M, Lankford H, Loeb LS, Wyckoff JH (2011). The influence of school administrators on teacher retention decisions. Am Educ Res J.

[CR17] Colbert BA (2004). The complex resource-based view: implications for theory and practice in strategic human resource management. Acad Manag Rev.

[CR18] Creswell JW (2012). Educational research: planning, conducting, and evaluating quantitative and qualitative research.

[CR19] Creswell JW, Miller DL (2000). Determining validity in qualitative inquiry. Theor Pract.

[CR21] Davies B (2003). Rethinking strategy and strategic leadership in schools. Educ Manag Admin Leadersh.

[CR22] Day C, Møller J, Nusche D, Pont B (2007). The Flemish approach to school leadership for systemic improvement. A case study report for the OECD activity improving school leadership.

[CR23] De Maeyer S, Rymenans R, Van Petegem P, Van den Bergh H, Rijlaarsdam G (2007). Educational leadership and pupil achievement: the choice of a valid conceptual model to test effects in school effectiveness research. Sch Eff Sch Improv.

[CR24] Department of Education (2009). Omzendbrief omtrent functiebeschrijving en evaluatie in het basisonderwijs [Letter to primary schools about job descriptions and teacher evaluation].

[CR25] Desimone LM, Porter AC, Garet MS, Yoon KS, Birman BF (2002). Effects of professional development on teachers’ instruction: results from a three-year longitudinal study. Educ Eval Policy Anal.

[CR26] Devos G, Vanderheyden K (2002). Attracting, developing and retaining effective teachers. Background report for Flanders.

[CR27] Devos G, Verhoeven J, Stassen K, Warmoes V (2004). Personeelsbeleid in Vlaamse scholen [Personnel policy in Flemish schools].

[CR28] Devos G, Van Petegem P, Vanhoof J, Declercq L, Delvaux E (2014). Evaluatie van het evaluatiesysteem voor leerkrachten in het basisonderwijs en het deeltijds kunstonderwijs [Evaluation of the evaluation system for teachers in primary education and part-time arts education].

[CR29] Devos G, Tuytens M, De Coninck K, Staelens E (2016). Uitdagingen voor personeelsbeleid: aanwerving en opdrachttoewijzing in Vlaamse basis- en secundaire scholen [Challenges for personnel policy: hiring and assignment in Flemish primary and secundary schools].

[CR30] Donaldson ML (2013). Principals’ approaches to cultivating teacher effectiveness: constraints and opportunities in hiring, assigning, evaluating and developing teachers. Educ Admin Q.

[CR31] European Commission (2013) Key data on teachers and school leaders in Europe. Eurydice Report. Publications Office of the European Union, Luxembourg

[CR32] Gilbert C, De Winne S, Sels L (2011). The influence of line managers and HR department on employees’ affective commitment. Int J Hum Resour Manag.

[CR34] Glick WH (1985). Conceptualizing and measuring organizational and psychological climate: pitfalls in multilevel research. Acad Manag Rev.

[CR35] Gould-Williams JS (2003). The importance of HR practices and workplace trust in achieving superior performance: a study of public-sector organizations. Int J Hum Resour Manag.

[CR36] Gratton L, Hope-Hailey V, Stiles P, Truss C (1999). Linking individual performance business strategy: the people process model. Hum Resour Manag.

[CR37] Guest D (1987). Human resources management and industrial relations. J Manag Stud.

[CR38] Hallinger P (2000) A review of two decades of research on the principalship using the principal instructional management rating scale. Paper presented at the annual meeting of the American Educational Research Association, Seattle, Washington

[CR39] Hallinger P (2003). Leading educational change: reflections on practice of instructional and transformational leadership. Camb J Educ.

[CR40] Hallinger P (2011). Leadership for learning: lessons from 40 years of empirical research. J Educ Admin.

[CR41] Hallinger P, Heck RH (1998). Exploring the principal’s contribution to school effectiveness: 1980–1995. Sch Eff Sch Improv.

[CR42] Hallinger P, Murphy J (1986). The social context of effective schools. Am J Educ.

[CR43] Horng E, Loeb S (2010). New thinking about instructional leadership. Phi Delta Kappan.

[CR44] Hosmer DW, Lemeshow S (2000). Applied logistic regression.

[CR45] Howell JM, Avolio BJ (1993). Transformational leadership, transactional leadership, locus of control, and support for innovation: key predictors of consolidated-business-unit performance. J Appl Psychol.

[CR46] Hoy W, Tarter C (1997). The road to open and healthy schools: a handbook for change.

[CR47] Hulpia H, Devos G, Rosseel Y (2009). Development and validation of scores on the distributed leadership inventory. Educ Psychol Meas.

[CR48] Huselid MA (1995). The impact of human resource management practices on turnover, productivity, and corporate financial performance. Acad Manage J.

[CR49] Huselid MA, Becker BE (1996). Methodological issues in cross-sectional and panel estimates of the Human Resource-firm performance link. Ind Relat.

[CR50] Johnson RB, Christensen LB (2008). Educational research: quantitative, qualitative, and mixed approaches.

[CR51] Johnson SM, Birkeland S, Kardos SM, Kauffman D, Liu E, Peske HG (2001) Retaining the next generation of teachers: the importance of school-based support. Harvard Education Letter

[CR52] Kepes S, Delery JE, Boxall P, Purcell J, Wright P (2007). HRM Systems and the problem of internal fit. Oxford handbook of human resource management.

[CR53] Kimball SM (2011). Principals as human capital managers at every school. Phi Delta Kappa.

[CR54] Koppich JE, Humphrey DC, Bland JA, Heenan B, McCaffery T, Ramage K, Stokes L (2013) California’s beginning teachers: The bumpy path to a profession. SRI International, J. Koppich and Associates, and Iverness Research, Menlo Park

[CR55] Kwan P (2009). Beginning teachers’ perceptions of school human resource practices. Asia Pac J Educ.

[CR56] Leisink P, Boselie P (2014) Strategisch HRM voor beter onderwijs: Een bijdrage aan de professionalisering van schoolleiders in het voorgezet onderwijs [Strategic HRM for better education. A contribution to principals’ professional development in secondary education]. Departement voor Bestuurs- en Organisatiewetenschap (USBO), Universiteit Utrecht

[CR57] Leithwood K (1992). The move toward transformational leadership. Educ Leadersh.

[CR58] Leithwood KA (1994). Leadership for school restructuring. Educ Admin Q.

[CR59] Leithwood K, Jantzi D (1999). The effects of transformational leadership on organizational conditions and student engagement with. J Educ Admin.

[CR60] Leithwood K, Jantzi D (2000). Transformational school leadership effects: a replication. Sch Eff Sch Improv.

[CR61] Leithwood K, Jantzi D (2006). Transformational school leadership for large-scale reform: effects on students, teachers, and their classroom practices. Sch Eff Sch Improv.

[CR62] Leithwood KA, Sun J (2012). The nature and effects of transformational school leadership: a meta-analytic review of unpublished research. Educ Admin Q.

[CR63] Leithwood K, Leonard L, Sharratt L (1998). Conditions fostering organizational learning in schools. Educ Admin Q.

[CR64] Lepak DP, Snell SA (1999). The human resource architecture: toward a theory of human capital allocation and development. Acad Manag Rev.

[CR65] Liu W, Lepak DP, Takeuchi R, Sims HP (2003). Matching leadership styles with employment modes: strategic human resource management perspective. Hum Resour Manag Rev.

[CR66] Louis K, Marks HM (1998). Does professional community affect the classroom? Teachers’ work and student experiences in restructuring schools. Am J Educ.

[CR67] Louis K, Dretzke B, Wahlstrom K (2010). How does leadership affect student achievement? Results from a national US survey. Sch Eff Sch Improv.

[CR68] Louis KS, Leithwood K, Wahlstrom KL, Anderson SE (2010). Investigating the Links to Improved Student Learning.

[CR69] Marks HM, Printy SM (2003). Principal leadership and school performance: an integration of transformational and instructional leadership. Educ Admin Q.

[CR70] Milanowski A, Kimball S, Curtis RE, Wurtzel J (2010). The principal as human capital manager: lessons from the private sector. Teaching talent: a visionary framework for human capital in education.

[CR71] Miles M, Huberman M (1994). Qualitative data analysis.

[CR72] Nehles A, van Riemsdijk M, Kok I, Looise J (2006). Implementing human resource management successfully: a first-line management challenge. Manag Revue.

[CR99] Odden A (2011). Strategic management of human capital in education. Improving instructional practice and student learning in schools.

[CR74] Onwuegbuzie AJ, Teddlie C, Tashakkori A, Teddlie C (2003). A framework for analyzing data in mixed methods research. Handbook of mixed methods in social and behavioral research.

[CR75] Papa F, Baxter I (2008). Hiring teachers in New York’s public schools: can the principal make a difference?. Leadersh Policy Sch.

[CR76] Patton M (1980). Qualitative evaluation methods.

[CR77] Purcell J, Hutchinson S (2007). Front-line managers as agents in the HRM-performance causal chain: theory, analysis and evidence. Hum Resour Manag J.

[CR78] Purcell J, Kinnie N, Swart J, Rayton B, Hutchinson S (2009). People management and performance.

[CR79] Rickards T (2006). The dilemmas of leadership.

[CR80] Ridder H-G, McCandless A (2010). Influences on the architecture of human resource management in nonprofit organizations: an analytical framework. Nonprof Volunt Sec Q.

[CR81] Ridder H-G, McCandless A, Piening EP (2012). The whole is more than the sum of its parts? How HRM is configured in nonprofit organisations and why it matters. Hum Resour Manag Rev.

[CR82] Robinson VMJ, Lloyd CA, Rowe KJ (2008). The impact of leadership on student outcomes: an analysis of the differential effects of leadership types. Educ Admin Q.

[CR83] Runhaar P, Runhaar H (2012). HR policies and practices in vocational education and training institutions: understanding the implementation gap through the lens of discourses HR policies and practices in vocational education and training institutions: understanding the implementation. Hum Resour Dev Int.

[CR84] Shatzer RH, Caldarella P, Hallam PR, Brown BL (2013). Comparing the effects of instructional and transformational leadership on student achievement: implications for practice. Educ Manag Admin Leadersh.

[CR85] Shen J, Leslie JM, Spybrook JK, Ma X (2012). Are principal background and school processes related to teacher job satisfaction? A multilevel study using schools and staffing survey 2003–04. Am Educ Res J.

[CR86] Smylie MA, Wenzel SA (2006). Promoting instructional improvement: a strategic human resource management perspective.

[CR87] Smylie MA, Miretzky D, Konkol P (2004). Rethinking teacher workforce development: a strategic human resource management perspective. Yearb Natl Soc Study Educ.

[CR88] Staelens E, Lesage H, Leunis M, Ballet K, Pauwels T (2014). Opleidingsnoden van beginnende directeurs [Training needs of beginning principals].

[CR89] TALIS (2008) The experience of new teachers. Results from TALIS 2008. OECD, Paris

[CR90] Vekeman E, Devos G, Valcke M (2015). Human resource architectures for new teachers in Flemish primary education. Educ Manag Admin Leadersh.

[CR91] Vermeeren B (2014). Variability in HRM implementation among line managers and its effect on performance: a 2-1-2 mediational multilevel approach. Int J Hum Resour Manag.

[CR92] Vermeeren B, Kuipers BS, Steijn AJ (2014). Does leadership style make a difference? Linking HRM, job satisfaction, and organizational performance. Rev Public Pers Admin.

[CR93] Wright PM, McMahan GC (1992). Theoretical perspectives for strategic human resource management. J Manag.

[CR94] Wright PM, Nishii LH (2007) Strategic HRM and organizational behavior: Integrating multiple levels of analysis. CAHRS Working Paper 07-03. Cornell University, School of Industrial and Labor Relations, Center for Advanced Human Resource Studies, Ithaca

[CR95] Wright PM, Snell SA (1998). Toward a unifying framework for exploring fit and flexibility in strategic human resources management. Acad Manag Rev.

[CR96] Wright PM, Dunford BB, Snell SA (2001). Human resources and the resource based view of firm. J Manag.

[CR97] Youngs P (2007). How elementary principals’ beliefs and actions influence new teachers’ experiences. Educ Admin Q.

[CR98] Zhu W, Chew IKH, Spangler WD (2005). CEO transformational leadership and organizational outcomes: the mediating role of human–capital-enhancing human resource management. Leadersh Q.

